# Prehospital fibrinolysis versus primary percutaneous coronary intervention in ST-elevation myocardial infarction: a systematic review and meta-analysis of randomized controlled trials

**DOI:** 10.1186/s13054-016-1530-z

**Published:** 2016-11-05

**Authors:** Vincent Roule, Pierre Ardouin, Katrien Blanchart, Adrien Lemaitre, Julien Wain-Hobson, Damien Legallois, Joachim Alexandre, Rémi Sabatier, Paul Milliez, Farzin Beygui

**Affiliations:** Department of Cardiology, Caen University Hospital, Avenue Cote de Nacre, 14033 Caen, France

**Keywords:** Primary percutaneous coronary intervention, Fibrinolysis, Prehospital, Mortality, Stroke

## Abstract

**Background:**

Primary percutaneous coronary intervention (PPCI) is the preferred reperfusion strategy in patients with ST-elevation myocardial infarction (STEMI), but its benefit over prehospital fibrinolysis (FL) is not clear.

**Methods:**

We performed a systematic review and meta-analysis of randomized controlled trials in which outcomes of patients with STEMI managed with FL early in the prehospital setting versus PPCI were compared.

**Results:**

Compared with PPCI, FL was consistently associated with similar rates of short-term (30–90 days) death (relative risk [RR] 0.94, 95 % CI 0.67–1.31) and cardiovascular death (RR 0.95, 95 % CI 0.64–1.4), a decreased risk of cardiogenic shock (RR 0.67, 95 % CI 0.48–0.95), and an increased risk of any stroke (RR 3.57, 95 % CI 1.39–9.17) and hemorrhagic stroke (RR 4.37, 95 % CI 1.25–15.26). FL was also associated with similar rates of 1-year mortality (RR 1.01, 95 % CI 0.75–1.34) and major bleeding (RR 1.31, 95 % CI 0.96–1.78) in comparison with PPCI, but with a notable level (*I* ^2^ index 30.5 % and 59.8 %) of heterogeneity among studies.

**Conclusions:**

Our study suggests that, compared with PPCI, FL performed in the early prehospital setting is associated with similar mortality rates, lower rates of cardiogenic shock, and higher rates of stroke in patients with STEMI. Although the number of studies comparing the two strategies is relatively low, our results support prehospital FL and transfer to hub percutaneous coronary intervention (PCI) centers as a valid alternative to PPCI, allowing potential limitation of resources allocated to developing proximity 24/7 PCI facilities.

**Electronic supplementary material:**

The online version of this article (doi:10.1186/s13054-016-1530-z) contains supplementary material, which is available to authorized users.

## Background

Primary percutaneous coronary intervention (PPCI) is considered the preferred reperfusion strategy in patients with ST-elevation myocardial infarction (STEMI), provided it can be performed expeditiously by an experienced team [1], based on studies comparing PPCI with in-hospital fibrinolysis (FL) [2]. International guidelines also underscore the objective of a total ischemic time <2 h in STEMI, which may not always be achievable if a PPCI strategy is chosen. Prehospital FL and direct transfer to a percutaneous coronary intervention (PCI)-capable center, which should be considered only when the estimated total ischemic time is >2 h after first medical contact in STEMI patients, is recommended over transfer for in-hospital FL when possible [3, 4].

Importantly, the relative benefit of PPCI over fibrinolytic therapy is time-dependent [5]. The benefit of PPCI over prehospital FL is not clear among patients managed early in the prehospital setting. The Comparison of primary Angioplasty and Pre-hospital fibrinolysis In acute Myocardial infarction (CAPTIM) trial [6] was the first large-scale trial comparing the two strategies. It showed that PPCI was not associated with lower mortality rates than prehospital FL. That study was terminated before reaching its target sample size, however. Researchers in the more recent Strategic Reperfusion Early after Myocardial Infarction (STREAM) trial [7] also reported similar rates of mortality between prehospital FL or PPCI. Both studies were undersized to assess a difference in mortality. In a real-life French nationwide registry of STEMI, prehospital FL was associated with reduced mortality in comparison with PPCI [8]. Hence, the benefit of allocating resources to developing proximity centers with 24/7 PCI facilities over prehospital FL and transfer to hub PCI centers may be questionable. The principal objective of the present systematic review and meta-analysis of randomized controlled trials was to compare prehospital FL and PPCI in terms of mortality.

## Methods

### Study selection

We conducted a systematic literature review by formal searches of the electronic databases MEDLINE (source PubMed) and the Cochrane Controlled Clinical Trials Register Database through January 2015. Relevant randomized controlled trials were identified by a combination of medical subject headings including the following terms: “myocardial infarction”, “acute myocardial infarction”, “STEMI”, “fibrinolytic therapy”, “fibrinolysis”, “thrombolysis”, “thrombolytic therapy”, “percutaneous coronary intervention”, “primary PCI”, and “primary angioplasty.” References from reviews and selected articles were also reviewed for potential relevant citations. Studies were selected by two independent reviewers (VR and PA).

We restricted our analysis to the trials that met all of the following inclusion criteria: (1) randomized controlled comparison between prehospital FL and PPCI, (2) in patients with STEMI managed in the prehospital setting early after symptom onset (<6 h), and (3) available data on mortality.

The primary outcome assessed in our analysis was mortality as reported in the principal publications. Other outcomes were recurrent myocardial infarction (MI), cardiogenic shock, stroke (hemorrhagic and ischemic), and the major bleeding as defined in each study. The meta-analysis of the primary composite endpoint of the studies was performed but should be considered only as exploratory because of the inclusion of different events in different studies and the subsequent risk of unpredictable sources of bias. Outcomes were based on the longest follow-up available for each study. We excluded studies that associated FL with glycoprotein IIb/IIIa inhibitors and studies with no clinical endpoint.

### Statistical analysis

The total numbers of patients experiencing or not the outcomes of interest in each arm extracted directly from the publications were used for the analyses. Results are presented as relative risks (RRs) with 95 % CIs. Outcomes from individual studies were combined using Mantel-Haenszel fixed effect and random-effects models. Heterogeneity across studies was evaluated by the Cochran’s Q statistic with a *p* value set at 0.1. The *I* ^2^ statistic was also taken into account, regardless of the *p* value. An *I* ^2^ ≥ 50 % was prespecified as the threshold considered too high to provide consistent analysis.

The random-effects model was considered for the primary analysis. A fixed effect model is also reported in figures, considered as a sensitivity analysis only. We also conducted a sensitivity analysis based on a study-by-study exclusion process. Tests were two-tailed, and a *p* value <0.05 was considered statistically significant. R software version 3.0.0 (2013-04-03) for Mac OS (R Foundation for Statistical Computing, Vienna, Austria) with the Meta package was used for the statistical analysis.

## Results

**Fig. 1:**
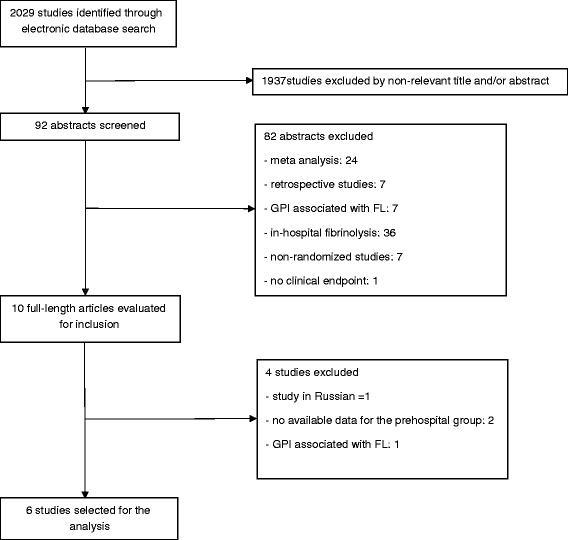
Flow diagram of meta-analysis trial selection. *GPI* Glycoprotein IIb/IIIa inhibitors, *FL* Fibrinolysis

Ultimately, three trials [6, 7, 9], comprising two long-term follow-up studies of the latter [10, 11] and a post hoc analysis of one trial [12], were selected for the meta-analysis. The review process is depicted in Fig. 1. The endpoints were collected at 30 days in the STREAM and CAPTIM studies and at 90 days in the Assessment of the Safety and Efficacy of a New Treatment Strategy with Percutaneous Coronary Intervention (ASSENT-4 PCI) study. The major characteristics of the patients of each study and the rates of the clinical endpoints are detailed in Table 1.

**Table 1:** Demographic characteristics of the patients and clinical endpoints of selected studies

	STREAM		CAPTIM		ASSENT-4 PCI	
	Prehospital FL (*n* = 944)	PPCI (*n* = 948)	Prehospital FL (*n* = 419)	PPCI (*n* = 421)	Prehospital FL (*n* = 163)	PPCI (*n* = 171)
Demographics						
Age, years	59.7 ± 12.4^a^	59.6 ± 12.5^a^	58 (49–69)^b^	58 (50–68)^b^	60 (51–69)^b^	
Age ≥75 years	134 (14.2 %)	121(12.8 %)	42 (10.0 %)	40 (9.5 %)	n/a	n/a
Female sex	194 (20.6 %)	208 (21.9 %)	74 (17.5 %)	78 (18.5 %)	19.5 %	
Diabetes	113/934 (12.1 %)	123/939 (13.1 %)	46 (11.1 %)	57 (13.5 %)	13.2 %	
Hypertension	434/930 (46.7 %)	414/932 (44.4 %)	141 (33.9 %)	146 (34.8 %)	43.4 %	
Dyslipidemia			212 (51.1 %)	215 (51.4 %)	35.9 %	
Previous PCI	60/942 (6.4 %)	83/944 (8.8 %)	22 (5.3 %)	18 (4.3 %)	9.4 %	
Previous CABG	2/944 (0.2 %)	3/946 (0.3 %)	0	5 (1.2 %)	1.2 %	
Previous MI	81/940 (8.6 %)	98/947 (10.3 %)	34 (8.2 %)	28 (6.7 %)	11.5 %	
Heart rate	74.9 ± 18.4	75.5 ± 18.1	76 (64–84)	75 (66–88)	n/a	n/a
Systolic blood pressure, mmHg	135.0 ± 22.7	135.9 ± 14.9	125 (110–140)	128 (111–140)	n/a	n/a
Anterior MI	453/942 (48.1 %)	431/946 (45.6 %)	166 (40.2 %)	178 (42.7 %)	45.8 %	
Median time delay (IQR), minutes						
Symptom onset to randomization, minutes	91 (68–132)	92 (65–132)	107 (76–158)	108 (76–162)	105 (75–172)	105 (70–160)
Symptom onset to start of reperfusion treatment: thrombolysis or balloon inflation	100 (75–143)	178 (135–230)	130 (95–180)	190 (149–255)	125 (90–185)	203 (154–258)
PCI	736/915 (80.4 %)	838/933 (89.9 %)	295 (70.4 %)	364 (86.5 %)	n/a	n/a
Endpoints						
Primary composite endpoint^c^	116/939 (12.4 %)	135/943 (14.3 %)	34 (8.2 %)	26 (6.2 %)	31/161 (19.3 %)	23/165 (13.9 %)
Death	43/939 (4.6 %)	42/946 (4.4 %)	16 (3.8 %)	20 (4.8 %)	5/163 (3.1 %)	7/171 (4.1 %)
Stroke	15/939 (1.6 %)	5/946 (0.5 %)	4 (1.0 %)	0	n/a	n/a
Reinfarction	23/938 (2.5 %)	21/944 (2.2 %)	15 (3.7 %)	7 (1.7 %)	n/a	n/a
Severe hemorrhage	70/939 (7.5 %)	47/946 (5 %)	2 (0.5 %)	8 (2.0 %)	13/163 (8.0 %)	11/171 (6.4 %)

*Abbreviations: CABG* Coronary artery bypass graft, *MI* Myocardial infarction, *n/a* Not available, *PCI* Percutaneous coronary intervention, *PPCI* Primary percutaneous coronary intervention, *ASSENT-4 PCI* Assessment of the Safety and Efficacy of a New Treatment Strategy with Percutaneous Coronary Intervention study, *CAPTIM* Comparison of primary Angioplasty and Pre-hospital fibrinolysis In acute Myocardial infarction trial, *STREAM* Strategic Reperfusion Early after Myocardial Infarction trial

^a^Mean

^b^Median (IQR)

^c^The primary composite endpoint was a composite of “death, shock, congestive heart failure or reinfarction at 30 days” for STREAM; “death, non-fatal reinfarction, and non-fatal disabling stroke in 30 days” for CAPTIM; and “mortality, shock, or congestive heart failure within 90 days” for ASSENT-4 PCI

**Fig. 2:**
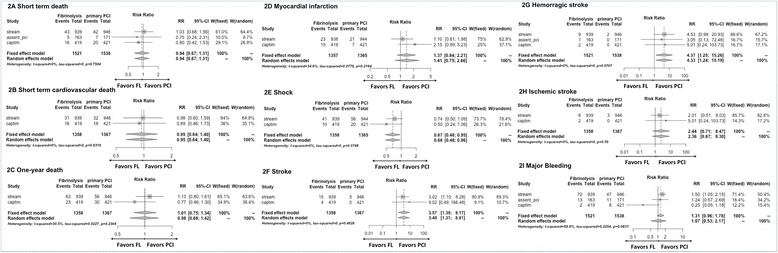
Forest plots of CAPTIM, STREAM and ASSENT-4 PCI studies comparing the effect of prehospital fibrinolysis with primary percutaneous coronary intervention on death (**a**), cardiovascular death (**b**), 1-year death (**c**), myocardial infarction (**d**), cardiogenic shock (**e**), stroke (**f**), hemorrhagic stroke (**g**), ischemic stroke (**h**), and major bleeding (**i**). *ASSENT-4 PCI* Assessment of the Safety and Efficacy of a New Treatment Strategy with Percutaneous Coronary Intervention study, *CAPTIM* Comparison of primary Angioplasty and Pre-hospital fibrinolysis In acute Myocardial infarction trial, *PCI* Percutaneous coronary intervention, *RR* Relative risk, *STREAM* Strategic Reperfusion Early after Myocardial Infarction trial

The meta-analysis showed consistently that the rates of short-term (30/90 days) death (RR 0.94, 95 % CI 0.67–1.31) (Fig. 2a) and cardiovascular death (RR 0.95, 95 % CI 0.64–1.4) (Fig. 2b) were similar between prehospital FL and PPCI. Similar results were also found for 1-year death (RR 1.01, 95 % CI 0.75–1.34) (Fig. 2c) and MI (RR 1.37, 95 % CI 0.84–2.21) (Fig. 2d) between the two strategies, but with nonsignificant but notable heterogeneity (*I* ^2^ index 30.5 % and 34.6 %).

Consistently, compared with PPCI, the risk of cardiogenic shock was lower (RR 0.67, 95 % CI 0.48–0.95) (Fig. 2e), but the risks of stroke (RR 3.57, 95 % CI 1.39–9.17) (Fig. 2f) and hemorrhagic stroke (RR 4.37, 95 % CI 1.25–15.26) (Fig. 2 g) were higher, in association with prehospital FL, whereas the risk of ischemic stroke (RR 2.44, 95 % CI 0.71–8.47) (Fig. 2 h) did not significantly differ between the two strategies. Finally, the risk of major bleeding (RR 1.31, 95 % CI 0.96–1.78) (Fig. 2i) was similar between the two strategies, but with a high level of heterogeneity between the studies (*I* ^2^ = 59.8 %).

Although the meta-analysis of the composite primary endpoints showed similar results between FL and PPCI (RR 0.99, 95 % CI 0.82–1.2), it should be considered only as exploratory because different endpoints composed the primary outcome of different studies. The sensitivity analyses by random-effects analysis (Fig. 2) and by excluding study by study (Additional file 1: Table S1) did not show any major change in the results of the principal analysis.

## Discussion

Our meta-analysis suggests that prehospital FL is associated with similar early and late mortality rates compared with PPCI in patients with STEMI managed early after symptom onset in the prehospital setting. Prehospital FL is also associated with a decreased risk of cardiogenic shock. However, it was consistently associated with an increased risk of stroke and hemorrhagic stroke.

In STEMI patients presenting in the first hours after symptom onset, PPCI is the recommended default reperfusion strategy if performed according to the recommended time limits [1]. Because most of these patients present to non-PPCI-capable hospitals, this reperfusion strategy may be a major challenge in many regions of the world [13]. Hence, recent trials have shown that prehospital FL appears to be a valuable alternative [6, 7, 9]. Our meta-analysis confirms that mortality rates and, less consistently, ischemic composite outcomes are similar between the two reperfusion strategies. This is concordant with real-life registries reporting comparable in-hospital [14] and even higher 1- and 5-year survival rates [8, 15] associated with prehospital FL compared with PPCI in similar populations. A major advantage of prehospital FL is the time gained from symptom onset to reperfusion (i.e., from symptom onset to start of reperfusion treatment with tenecteplase or alteplase in the FL group and first balloon inflation in the PPCI group), ranging from 60 to 78 minutes in the studies included in our analysis [6, 7, 9]. Such times are reported in countries where the density of centers with PPCI facilities is relatively high. Hence, it may be speculated that in many other regions, such times are even longer. Indeed, the time from symptom onset to reperfusion is of critical importance for myocardial salvage [16]. The use of a single-bolus fibrinolytic therapy as described in the STREAM and ASSENT-4 PCI trials [7, 9], but not in the CAPTIM trial [6], may be preferable because of its quick and easy administration. The use of continuous intravenous infusion of alteplase may also explain the relatively shorter difference of time from symptom onset to reperfusion therapy between the PPCI and FL groups in the CAPTIM trial [6] than in other trials.

Prehospital FL is recommended [1, 3] in association with early routine angiography, and PCI if needed. In the studies included in our analysis, patients were systematically transferred to PCI-capable centers and had high rates of PCI (70.4 %, 80.4 %, and 86.7 %, respectively, in the FL groups of the CAPTIM, STREAM, and ASSENT-4 PCI trials). The Which Early ST-elevation myocardial infarction Therapy (WEST) study [17] researchers previously showed that a strategy of early FL (only 18 % prehospital) coupled with routine early invasive management results in similar rates of death and recurrent MI compared with PPCI. Unlike these favorable results, the large Swedish registry [18] reported lower mortality rates in association with PPCI than both in-hospital and prehospital FL. Such discordance is probably explained by the low rates of PCI in the prehospital FL group (47.3 % of PCI or coronary artery bypass graft [CABG] within 14 days) in this registry. The optimal delay between successful FL and PCI is also a critical issue. Compared with PPCI, prehospital tenecteplase-facilitated PCI was associated with increased rates of reinfarction and trends toward higher rates of mortality [9] and increased infarct size [19]. Such results are explained by the paradoxical FL-induced platelet hyperreactivity and thrombin-induced platelet activation within the early hours after FL [20]. Hence, the time window of 3–24 h after successful FL is recommended [1] and explains the excellent results of the pharmacoinvasive strategy, as demonstrated in our meta-analysis.

Trends toward lower rates of cardiogenic shock occurrence with prehospital FL were noted in the CAPTIM [6] and STREAM [7] trials as well as in the WEST study [17]. Our meta-analysis shows a consistent and significant reduction of rates of cardiogenic shock complicating STEMI in association with prehospital FL compared with PPCI. This is also consistent with the fact that the most frequent cause of death was cardiogenic shock in the trials [6, 9]. In the CAPTIM trial [6], all shocks that occurred during the transport to the hospital were observed in the PPCI group, suggesting that the 1-h additional delay in the PPCI compared with prehospital FL may be responsible for the higher cardiogenic shock rates.

However, our meta-analysis highlights the consistent relative increase of the risks of stroke and hemorrhagic stroke in association with prehospital FL compared with PPCI. This finding may be tempered by the low absolute rates of stroke in each study, however, potentially explained by the limited proportion of elderly patients included. The increased risk of intracranial bleeding in patients ≥75 years old in the STREAM study [7] led to a dose reduction of tenecteplase in such patients. After the subsequent protocol amendment, no cases of intracranial hemorrhage were reported, and rates of mortality significantly dropped in the FL arm [11]. Moreover, rates of major bleeding did not significantly differ between the two strategies in our analysis.

The time from symptom onset to first medical contact as well as patient characteristics should be taken into account before selecting the reperfusion strategy [3]. In the studies selected in our analysis, patients were included if they presented within 3–6 h of symptom onset. However, the benefit of prehospital FL may be even more important in the first 2 h after symptom onset, as it has previously been reported to be associated with an improved 1-year survival compared with PPCI (2.8 % versus 6.9 %, respectively; *p* = 0.021) and low rates of intracranial hemorrhage (0.3 %) [21]. The patient’s risk profile, stratified by age and infarct location (anterior versus other), is also of importance in selecting the reperfusion strategy. In a large North American registry including 192,509 patients [5], the mean PCI-related time delay compared with FL, where mortality rates of the two reperfusion strategies were comparable, was calculated as 114 minutes in general. This delay was reduced to only 40 minutes in patients <65 years old presenting with an anterior MI within 2 h of symptom onset. In such patients with an extensive area of myocardium at risk, low risk of intracranial hemorrhage, and high chance of successful FL [22], a rapid and efficient restoration of flow with prehospital FL may improve myocardial salvage and outcome.

### Study limitations

Our meta-analysis was not performed on individual patients’ data. Only few randomized trials, all included in the present analysis, have compared prehospital FL with PPCI. Aggregate data can be misleading in cases of few available studies, especially for the exploration of differences across subgroups. The included studies have a certain degree of clinical heterogeneity, which is mirrored by the statistical inconsistency found across some of the outcomes (1-year mortality, MI, major bleeding). We acknowledge this limitation of our study; however, the results are highly consistent for some major endpoints (30-/90-day and cardiovascular mortality, shock, stroke, hemorrhagic stroke), and the sensitivity analyses support the robustness of our results.

Owing to the low number of studies, which is a limitation but also a justification of the meta-analysis, confidence intervals for events with low occurrence rates (e.g., hemorrhagic stroke) are relatively large. Our conclusions do not apply to patients excluded from the studies, particularly those with symptom onset to first medical contact time >6 h, cardiogenic shock, a previous CABG, or any contraindication to FL. Finally, half-dose fibrinolytic therapy in patients >75 years old, associated with a reduction of stroke rates in the STREAM trial, was not applied in other studies. Whether such dose adaptation may further improve the results of fibrinolytic therapy needs to be tested by adequate studies.

## Conclusions

Our analysis suggests that prehospital FL is associated with similar early and late mortality rates compared with PPCI in patients with STEMI managed early after symptom onset in the prehospital setting. Prehospital FL was associated with a decreased risk of cardiogenic shock but an increased risk of stroke. Prehospital FL appears to be a valuable alternative to PPCI. Pharmacoinvasive strategies including prehospital FL and transfer to hub PCI centers may allow reduction of the cost allocated to developing proximity centers with 24/7 PCI facilities while providing similarly efficient reperfusion therapy.

## Additional file

Additional file 1: Table S1. Sensitivity analysis (by excluding study by study). (DOCX 14 kb)

## Abbreviations

ASSENT-4 PCI: Assessment of the Safety and Efficacy of a New Treatment Strategy with Percutaneous Coronary Intervention study
CABG: Coronary artery bypass graft
CAPTIM: Comparison of primary Angioplasty and Pre-hospital fibrinolysis In acute Myocardial infarction trial
FL: Fibrinolysis
GPI: Glycoprotein IIb/IIIa inhibitors
MI: Myocardial infarction
n/a: Not available
PCI: Percutaneous coronary intervention
PPCI: Primary percutaneous coronary intervention
RR: Relative risk
STEMI: ST-elevation myocardial infarction
STREAM: Strategic Reperfusion Early after Myocardial Infarction trial
WEST: Which Early ST-elevation myocardial infarction Therapy study
