# Supplementary material for: Prehospital fibrinolysis versus primary percutaneous coronary intervention in ST-elevation myocardial infarction: a systematic review and meta-analysis of randomized controlled trials
Source: Crit Care. 2016 Nov 5;20:359. doi: 10.1186/s13054-016-1530-z (PMC5097407; doi:10.1186/s13054-016-1530-z)
Supplement: Additional file 1: Table S1. — Sensitivity analysis (by excluding study by study). (DOCX 14 kb) [file 13054_2016_1530_MOESM1_ESM.docx]

**Additional file 1 Table S1:** Sensitivity analysis (by excluding study by study)

|  |  | All studies included | STREAM excluded | CAPTIM excluded | ASSENT-PCI excluded |
| --- | --- | --- | --- | --- | --- |
| Death | Fixed effect | 0.94 [0.67 ;1.31] | 0.79 [0.24 ;2.31] | 0.99[0.67 ;1.46] | 0.96[0.68 ;1.36] |
|  | Random effect | 0.94 [0.67 ;1.31] | 0.79 [0.24 ;2.31] | 0.99[0.67 ;1.47] | 0.96[0.68 ;1.36] |
| CV death | Fixed effect | 0.95 [0.64 ;1.40]* | - | - | 0.95 [0.64 ;1.40] |
|  | Random effect | 0.95 [0.64 ;1.40] * | - | - | 0.95 [0.64 ;1.40] |
| 1-year death | Fixed effect | 1.01 [0.75 ;1.34] * | - | - | 1.01 [0.75 ;1.34] |
|  | Random effect | 0.98 [0.68;1.42] * | - | - | 0.98 [0.68;1.42] |
| MI | Fixed effect | 1.37 [0.84 ;2.21] * | - | - | 1.37 [0.84 ;2.21] |
|  | Random effect | 1.41 [0.75 ;2.66] * | - | - | 1.41 [0.75 ;2.66] |
| Shock | Fixed effect | 0.67 [0.48 ;0.95] * | - | - | 0.67 [0.48 ;0.95] |
|  | Random effect | 0.68 [0.64 ;0.96] * | - | - | 0.68 [0.64 ;0.96] |
| Stroke | Fixed effect | 3.57 [1.39 ;9.17] * | - | - | 3.57 [1.39 ;9.17] |
|  | Random effect | 3.40 [1.31 ;8.81] * | - | - | 3.40 [1.31 ;8.81] |
| Hemorrhagic stroke | Fixed effect | 4.37 [1.25 ;15.26] | 4.03 [0.46 ;35.66] | 4.24 [1.07 ;16.67] | 4.63 [1.18 ;18.13] |
|  | Random effect | 4.33 [0.71 ;8.47] | 3.95 [0.44 ;35.31] | 4.21 [1.06 ;16.68] | 4.63 [1.18 ;18.12] |
| Ischemic stroke | Fixed effect | 2.44 [0.67 ;8.30] * | - | - | 2.44 [0.67 ;8.30] |
|  | Random effect | 2.36 [1.24 ;15.19] * | - | - | 2.36 [1.24 ;15.19] |
| Major bleeding | Fixed effect | 1.31 [0.96 ;1.78] | 0.82[0.42;1.58] | 1.45 [1.05 ;2.01] | 1.32[0.94;1.85] |
|  | Random effect | 1.07 [0.53 ;2.17] | 0.64[0.14;3.05] | 1.45 [1.05 ;2.01] | 0.72[0.13;4.08] |

* Results only available for CAPTIM and STREAM
